# HARM-YOLO11n: A Lightweight Real-Time Object Detection Framework for Robust Classroom Behavior Monitoring

**DOI:** 10.3390/s26154770

**Published:** 2026-07-27

**Authors:** Zihang Zhang, Yonghua Mao, Yingcang Ma, Xiaolin Gui

**Affiliations:** 1 School of Computer Science, Xi’an Polytechnic University, Xi’an 710048, China; 42309310212@stu.xpu.edu.cn; 2 School of Science, Xi’an Polytechnic University, Xi’an 710048, China; mayingcang@xpu.edu.cn; 3 School of Electronic and Information Engineering, Xi’an Jiaotong University, Xi’an 710049, China

**Keywords:** object detection, classroom behavior analysis, lightweight models, feature refinement, position-adaptive multi-scale fusion, class imbalance

## Abstract

Real-time classroom behavior monitoring is an important component of intelligent education systems, yet it remains challenging due to dense student layouts, frequent occlusions, and small-scale behavior targets. To address these challenges, this paper proposes Hierarchical Adaptive Re-parameterized Multi-scale YOLO11n (HARM-YOLO11n), a lightweight object detection framework based on YOLO11n for classroom behavior analysis. The proposed framework integrates structural re-parameterized feature enhancement (SRFE), a spatial-channel adaptive module (SCAM), position-adaptive multi-scale fusion (PAMSF), and IoU-adaptive soft weight (IASW) to improve feature representation, multi-scale interaction, and optimization behavior. SRFE enhances feature extraction through structural re-parameterization, the SCAM reduces redundant feature responses via selective spatial-channel computation, PAMSF performs adaptive multi-scale feature aggregation, and IASW introduces localization-aware sample weighting during training. Experiments on POCO-Dataset, SCB-Dataset3, and STBD-08 show that HARM-YOLO11n achieves improved detection performance compared with representative lightweight detectors while maintaining real-time inference efficiency. Multi-dataset evaluations further indicate stable performance under different data distributions. The results suggest that the proposed framework provides a practical approach for classroom behavior detection in resource-constrained educational environments.

## 1. Introduction

The increasing deployment of intelligent education systems has driven demand for reliable real-time classroom behavior understanding. Vision-based sensing systems are expected to provide continuous and structured behavioral feedback for educational analytics and classroom management [1,2]. Compared with manual observation, camera-based solutions offer scalable perception capabilities in complex classroom environments [3,4,5,6,7].

However, classroom behavior recognition [8,9] remains fundamentally challenging due to coupled visual ambiguities. Dense spatial configurations, fine-grained behavioral categories, and strong appearance similarity jointly induce occlusion, scale inconsistency, and feature ambiguity. These factors are inherently interdependent, leading to unstable representation learning in practical scenarios [10,11].

One-stage detectors, particularly the YOLO family [12,13,14,15], provide a strong accuracy–efficiency trade-off and are widely adopted in real-time vision systems. However, their performance degrades when multiple degradation factors co-occur, as standard architectures typically decouple feature representation, interaction, and optimization.

This limitation arises from a structural mismatch between independent module design and coupled scene degradation. In classroom environments, occlusion, scale variation, and class imbalance are not isolated factors but jointly influence feature extraction and decision consistency. This coupling introduces an interdependent optimization challenge, where improving one aspect (e.g., feature strength) may not translate to overall gains without simultaneously addressing the others. This motivates the need for a unified modeling strategy that jointly addresses representation quality, multi-scale interaction, and optimization stability within a lightweight framework.

To this end, we propose Hierarchical Adaptive Re-parameterized Multi-scale YOLO11n (HARM-YOLO11n), a unified detection framework that integrates re-parameterized feature enhancement, adaptive feature interaction, multi-scale fusion, and localization-aware optimization within a single architecture. While individual building blocks are adapted from established techniques—structural re-parameterization (inspired by RepVGG [16]), channel attention (SE [17]), partial convolution (PConv [18]), adaptive spatial fusion (ASFF [19]), and slide weighting (YOLO-FaceV2 [20])—the key novelty lies in (i) their joint integration targeting the coupled degradation factors specific to dense classroom scenarios, and (ii) the domain-specific adaptations introduced to each component for robustness under occlusion, scale variation, and class imbalance within a lightweight budget.

The main contributions are summarized as follows:

- We formulate classroom behavior detection as a coupled optimization problem and propose a unified lightweight framework for real-time deployment.
- We introduce a re-parameterized feature enhancement strategy that improves occlusion robustness without increasing inference cost.
- We design an adaptive feature interaction mechanism to suppress redundancy and improve representation selectivity in dense scenes.
- We develop a position-aware multi-scale fusion strategy for improved small-object modeling under scale variation.
- We propose a localization-aware optimization scheme that stabilizes training under ambiguous supervision signals.

## 2. Related Work

Classroom behavior detection has been studied from three perspectives: detection architectures, efficient representation learning, and feature interaction with optimization strategies.

Early approaches relied on handcrafted features or two-stage detectors such as Faster R-CNN [21], which achieve high accuracy but suffer from limited efficiency. With the rise of deep learning, one-stage detectors, particularly YOLO-based models [6,13,14,15], have become the dominant paradigm for real-time applications due to their favorable efficiency–accuracy balance.

Recent efforts improve performance through multi-scale feature modeling, attention mechanisms, and transformer-based global reasoning [22,23,24]. In parallel, vision-based educational sensing systems have been widely explored for classroom analytics [25,26,27]. However, these methods typically assume partial independence among challenges, which limits robustness when occlusion, scale variation, and fine-grained ambiguity occur simultaneously.

To improve efficiency, lightweight design techniques such as structural re-parameterization and attention-based feature recalibration have been widely adopted [16,17,28]. Nevertheless, these methods primarily enhance feature quality locally without explicitly modeling cross-component interactions under coupled scene degradation.

Beyond representation learning, multi-scale fusion [29] and optimization-aware learning have been investigated to improve robustness [19,23]. In parallel, several classroom-specific detectors have been proposed. Tang et al. [22] augment YOLOv5 with multi-scale feature fusion and attention, while Wang et al. [23] integrate squeeze-and-excitation attention into YOLOv5 for multi-student classroom behavior recognition. SmartLite-YOLO [30] combines a high-frequency enhanced residual block with focal modulation and dynamic upsampling and removes the large-object detection head to reduce computational overhead. YOLOv8n_BT [31] incorporates a bi-level routing attention mechanism into YOLOv8n to better handle occluded and small targets. YOLO-CBR [32] introduces dual-stage routing attention and multi-scale optimization on top of YOLOv11n for classroom behavior recognition.

Despite these advances, existing classroom-specific methods share a common limitation: they typically enhance detection through a single design dimension—either architecture, attention, or optimization—while treating feature extraction, fusion, and training objectives as independent modules. In contrast, occlusion, scale variation, and class imbalance in dense classroom scenes are inherently coupled, meaning that isolated improvements in one aspect may not translate to overall gains. HARM-YOLO11n differs from prior work by jointly addressing feature enhancement (SRFE), spatial-channel refinement (SCAM), multi-scale fusion (PAMSF), and localization-aware optimization (IASW) within a unified framework, where the four components cooperate rather than operate independently.

## 3. Proposed Method

Classroom behavior detection in real-world scenarios is challenged by dense occlusion, scale variation, feature redundancy, and class imbalance. These factors are highly coupled, making it difficult for lightweight detectors to achieve stable performance under real-time constraints.

To address the aforementioned challenges, HARM-YOLO11n is constructed upon YOLO11n and integrates four complementary components: structural re-parameterized feature enhancement (SRFE), a spatial-channel adaptive module (SCAM), position-adaptive multi-scale fusion (PAMSF), and IoU-adaptive soft weight (IASW). These components jointly enhance feature representation, adaptive feature interaction, multi-scale aggregation, and localization-aware optimization. For brevity, the abbreviations SRFE, SCAM, PAMSF, and IASW are used throughout the remainder of this paper.

**Figure 1:**
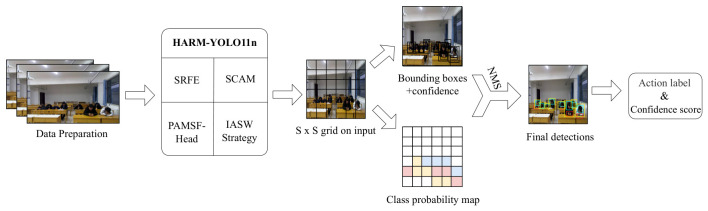
Overview of HARM-YOLO11n. The framework consists of SRFE-based feature extraction, SCAM-based feature refinement, PAMSF-based multi-scale fusion, and IASW-based optimization. SRFE, SCAM, PAMSF, and IASW denote Structural Re-parameterized Feature Enhancement, Spatial-Channel Adaptive Module, Position-Adaptive Multi-Scale Fusion, and IoU-Adaptive Soft Weight, respectively. The colored boxes denote different feature processing modules and bounding box predictions, while different colors in the class probability map represent distinct behavior categories and confidence scores.

As illustrated in Figure 1, HARM-YOLO11n adopts a progressive feature processing paradigm. SRFE first enhances backbone representations, the SCAM refines spatial-channel dependencies, and PAMSF performs cross-scale feature aggregation. During training, IASW adaptively reweights samples based on localization quality measured by IoU, improving optimization stability.

**Figure 2:**
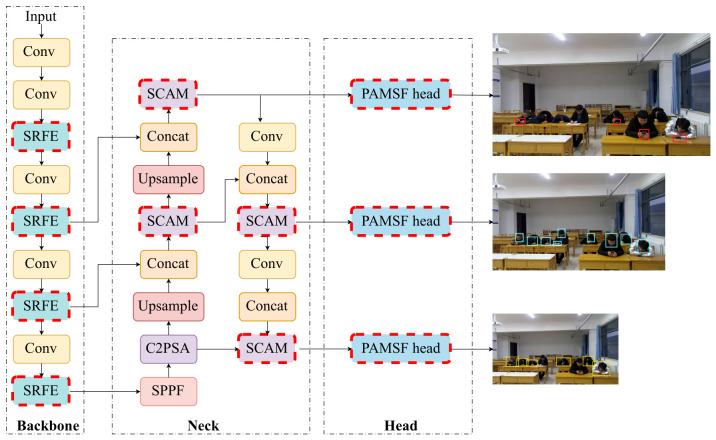
Architecture of HARM-YOLO11n integrating SRFE backbone, SCAM refinement module, PAMSF detection head, and IASW training strategy. Cyan and blue boxes denote the proposed core modules (SRFE, SCAM, and PAMSF head), while orange boxes represent standard YOLO11 structural components.

The overall architecture is shown in Figure 2.

### 3.1. Structural Re-Parameterized Feature Enhancement (SRFE)

To mitigate feature degradation caused by occlusion and fragmented visibility in dense classroom scenes, we introduce structural re-parameterized feature enhancement (SRFE) into the YOLO11n backbone.

SRFE is built upon a cross-stage partial (CSP) structure [33], where the standard bottleneck is replaced by a re-parameterized bottleneck (RepB). RepB adopts a multi-branch design during training and is equivalently transformed into a single convolution during inference via batch-normalization folding, enabling training-inference decoupling without additional deployment cost.

Each RepB contains three branches: a 3×3 convolution, a 1×1 convolution, and an identity mapping. Each branch is followed by batch normalization. Given input feature X, the training-stage formulation is

(1) $$Y_{\mathrm{train}}=\mathrm{BN}\left(W_{3\times 3}*X\right)+\mathrm{BN}\left(W_{1\times 1}*X\right)+\mathrm{BN}\left(X\right),$$

where $W_{3\times 3}$ and $W_{1\times 1}$ denote convolution kernels of different receptive fields.

**Figure 3:**
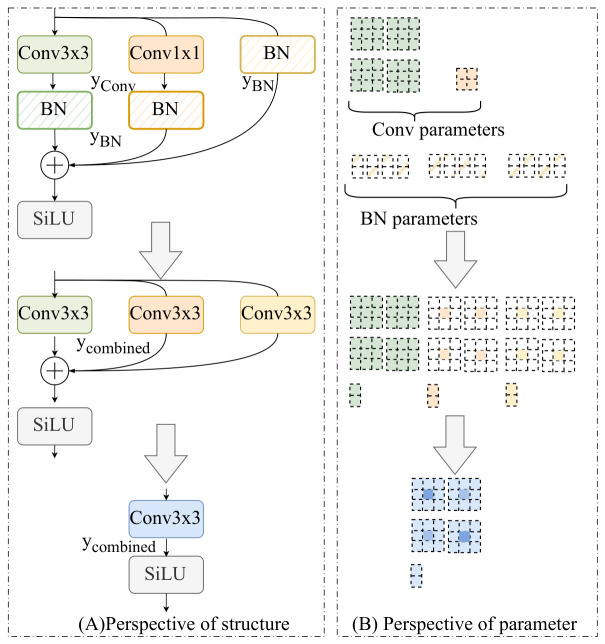
Re-parameterization in SRFE. Multi-branch training structure is fused into a single convolution during inference via batch-normalization folding. Green, orange, and yellow represent the 3×3 convolution, 1×1 convolution, and batch normalization branches/parameters during training, respectively, while blue denotes the equivalent merged 3×3 convolution during inference.

During inference, all branches are merged into a single equivalent convolution, preserving representational capacity while eliminating structural overhead (Figure 3).

To further enhance channel selectivity, a squeeze-and-excitation (SE) mechanism [17] is incorporated. Global average pooling generates channel descriptors, which are processed by a lightweight gating network to produce adaptive channel weights for feature recalibration.

**Figure 4:**
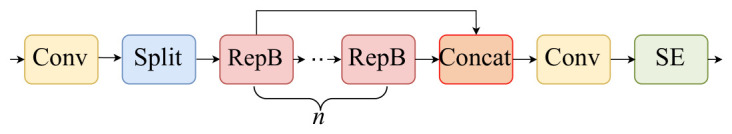
SRFE module combining RepB-based structural re-parameterization and SE-based channel recalibration within a CSP framework.

The SRFE module structure is illustrated in Figure 4. SRFE enhances representation robustness under occlusion while preserving inference efficiency through structural re-parameterization.

### 3.2. Spatial-Channel Adaptive Module (SCAM)

In dense classroom scenarios, feature redundancy arises frequently due to similar appearance patterns among adjacent subjects and shared background activations. Under lightweight deployment constraints, uniformly processing all channels leads to unnecessary computation and suboptimal feature utilization.

To address this issue, we introduce the spatial-channel adaptive module (SCAM) in the neck network. The SCAM integrates partial convolution (PConv) [18] into the C3k2 bottleneck of the cross-stage partial (CSP) framework [33], enabling selective spatial computation across channels. Here, C3k2 denotes the lightweight bottleneck structure adopted in YOLO11n, consisting of stacked convolutional blocks and partial feature aggregation for efficient feature extraction.

**Figure 5:**
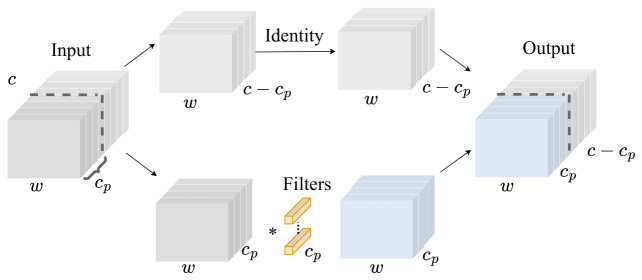
Partial Convolution (PConv). Spatial convolution is applied to a subset of channels, while the remaining channels are preserved via identity mapping. The symbol ∗ denotes the convolution operation.

As illustrated in Figure 5, PConv partitions the input feature channels into two subsets, where only a fraction is processed by spatial convolution while the remainder is propagated through identity mapping. The channel selection ratio is defined as

(2) $$r=\frac{c_{p}}{c},$$

where *c* and $c_{p}$ denote the total number of channels and the number of channels undergoing spatial convolution, respectively.

Accordingly, the computational complexity of the convolutional branch is approximately reduced in proportion to *r*:

(3) $${\mathrm{FLOPs}}_{\mathrm{PConv}}\propto r\cdot {\mathrm{FLOPs}}_{\mathrm{Conv}}.$$

However, the overall network complexity is jointly determined by backbone extraction, neck aggregation, and detection head computation. Therefore, the total inference cost does not scale strictly linearly with *r*.

Within the SCAM, channel-partitioned features are processed via a hybrid pathway: the selected subset undergoes convolution followed by batch normalization, while the remaining channels are directly forwarded. The two branches are subsequently concatenated and fused using a pointwise convolution to enable cross-channel interaction and restore representation consistency.

In this work, r=1/4 is empirically selected to achieve a balanced trade-off between representational capacity and computational efficiency.

**Figure 6:**
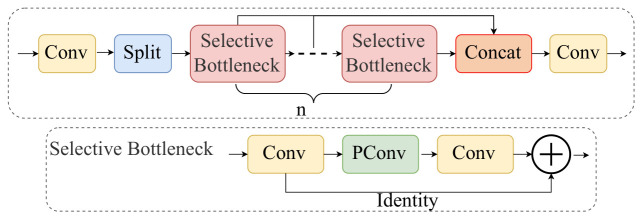
Architecture of SCAM. PConv is embedded within a C3k2 bottleneck under a CSP framework for efficient feature refinement.

The structure of the SCAM is shown in Figure 6. Overall, the SCAM introduces adaptive spatial computation at the channel level, improving feature utilization efficiency while maintaining compatibility with lightweight deployment requirements.

### 3.3. Position-Adaptive Multi-Scale Fusion (PAMSF)

In classroom scenes captured from fixed viewpoints, significant scale variations exist across spatial regions. Objects at different distances from the camera exhibit heterogeneous feature distributions, making fixed fusion strategies in conventional feature pyramid networks suboptimal.

To address this issue, we propose a position-adaptive multi-scale fusion (PAMSF) module, inspired by the adaptive spatial feature fusion (ASFF) mechanism [19]. Unlike conventional fixed-weight fusion, PAMSF dynamically assigns fusion weights conditioned on local feature context, allowing different spatial regions to selectively emphasize different semantic scales.

**Figure 7:**
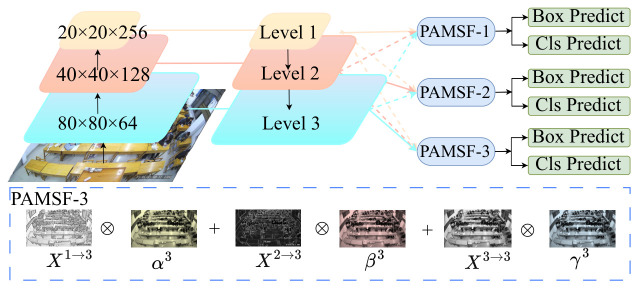
Architecture of PAMSF. The module performs position-adaptive multi-scale fusion via lightweight attention over pyramid features, enabling spatially varying scale selection.

As illustrated in Figure 7, PAMSF performs position-wise fusion across pyramid levels. The fused feature at spatial location (i,j) and level *l* is formulated as

(4) $$y_{ij}^{l}=\alpha_{ij}^{l}x_{ij}^{1\to l}+\beta_{ij}^{l}x_{ij}^{2\to l}+\gamma_{ij}^{l}x_{ij}^{3\to l},$$

where $x_{ij}^{k\to l}$ denotes the aligned feature from pyramid level *k* to level *l*.

The fusion weights satisfy a normalization constraint:

(5) $$\alpha_{ij}^{l}+\beta_{ij}^{l}+\gamma_{ij}^{l}=1,$$

ensuring a stable convex combination of multi-scale features.

The weights are generated via a lightweight attention function:

(6) $$\left[\alpha_{ij}^{l},\beta_{ij}^{l},\gamma_{ij}^{l}\right]=\mathrm{Softmax}\left(\lambda_{ij}^{l}\right),$$

where $\lambda_{ij}^{l}$ is computed from channel-compressed feature representations to reduce computational overhead while preserving spatial sensitivity.

Compared with fixed fusion schemes, PAMSF introduces explicit spatial adaptability in feature aggregation, enabling region-specific scale selection and improving robustness under scale-variant and occluded classroom scenarios.

### 3.4. IoU-Adaptive Soft Weight (IASW)

Besides architectural design, optimization behavior also influences detection performance in classroom scenarios characterized by class imbalance, partial occlusion, and localization ambiguity. Under such conditions, positive samples exhibit substantial variation in localization quality, yet conventional classification losses assign equal emphasis to all positive samples regardless of their regression accuracy.

To address this issue, we propose an IoU-adaptive soft weight (IASW) strategy, inspired by the slide loss introduced in YOLO-FaceV2 [20]. IASW assigns sample weights based on localization quality relative to the batch mean μ:

(7) $$w\left(\mathrm{IoU}\right)=\left\{\begin{array}{cc} 1, & \mathrm{IoU}\leq \mu -\Delta ,\\ \exp(1-\mu ), & \mu -\Delta <\mathrm{IoU}<\mu ,\\ \exp\left(1-\mathrm{IoU}\right), & \mathrm{IoU}\geq \mu , \end{array}\right.$$

where Δ is a margin hyperparameter set to 0.1. The three regions serve distinct roles. Region 1 (IoU≤μ−Δ) assigns unit weight to low-quality samples, preserving their learning signal without amplification. Region 2 (μ−Δ<IoU<μ) covers samples in a narrow transitional band below the batch mean. Since individual IoU values in this band are close to μ and subject to high measurement noise, IASW assigns a sample-independent constant exp(1−μ) controlled by the batch-level statistic. This avoids overfitting to unreliable per-sample IoU while retaining batch-adaptive emphasis: early in training when μ is low, these borderline samples receive a collective boost, which naturally decays as localization improves. Region 3 (IoU≥μ) weights above-average samples by exp(1−IoU), which peaks at moderate IoU (e.g., IoU=0.7⇒w=1.35) and approaches unity for near-perfect detections, preventing well-converged samples from dominating the gradient.

IASW differs from existing reweighting strategies in three key aspects. Focal loss [34] down-weights easy samples based on classification confidence $p_{t}$, but treats all positive samples uniformly with respect to localization quality—a well-localized box and a poorly localized one receive the same weight if their classification scores are similar. Quality focal loss (QFL) [35] extends focal loss by supervising the joint classification–IoU score, yet applies sample-level weighting without considering the batch-level localization distribution. Varifocal loss (VFL) [36] weights positive samples by their IoU score directly, assigning larger weight to high-IoU samples. In contrast, IASW introduces batch-adaptive thresholding via μ, which partitions samples into three regions relative to the current training state. The key distinction lies in Region 2: rather than relying on individual IoU values that are unreliable near the batch mean, IASW pools borderline samples under a group-level weight that evolves with training progress. This design is particularly suited to dense classroom scenes, where annotation boundaries are inherently ambiguous and per-sample IoU can fluctuate significantly. Experimental comparisons with these loss functions are provided in Section 4.

## 4. Experiments

This section evaluates the proposed HARM-YOLO11n framework for classroom behavior detection. The experimental evaluation covers dataset descriptions and implementation details, followed by ablation studies, quantitative comparisons, qualitative analysis, and multi-dataset evaluation.

### 4.1. Datasets and Implementation Details

Experiments are conducted on three publicly available classroom behavior datasets: POCO-Dataset, SCB-Dataset3, and STBD-08. These datasets feature dense classroom layouts, frequent occlusions, and significant object-scale variations, providing representative conditions for real-world classroom behavior detection.

**Figure 8:**
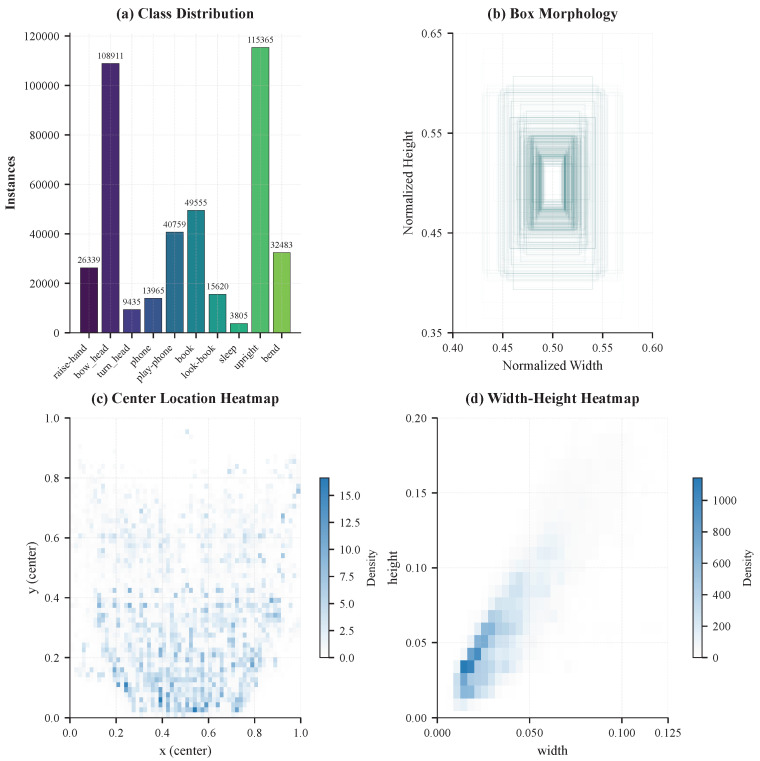
Distribution of bounding-box scales in the POCO-Dataset, showing a high proportion of small targets in dense classroom scenes.

The POCO-Dataset [37] contains 1903 RGB images with 137,960 annotated instances across ten behavior categories. As illustrated in Figure 8, a large proportion of annotated objects occupy small-scale regions, making accurate localization challenging under standard detection settings.

The SCB-Dataset3 [38] consists of 5015 images covering three behavior categories: hand-raising, reading, and writing. It exhibits substantial variations in viewpoint, illumination, and partial occlusion across different classroom environments.

The STBD-08 [39] dataset contains 8844 images with 267,888 annotated instances across eight behavior categories, including writing, reading, listening, standing, talking, guiding, turning around, and hand-raising. Compared with the other datasets, STBD-08 exhibits higher annotation density and more complex spatial interactions, making it suitable for evaluating dense-scene detection performance.

For dataset partitioning, the POCO-Dataset is randomly divided into training, validation, and testing sets with a ratio of 60%, 20%, and 20%, respectively, following the protocol of the original study. Since SCB-Dataset3 and STBD-08 do not provide official fixed partition files, we generate their training, validation, and testing splits using a fixed random seed. The same partition strategy is applied to all compared methods to ensure fair evaluation.

All experiments are implemented in PyTorch 2.3.0 and conducted on a workstation equipped with an NVIDIA RTX 4090 GPU (24 GB VRAM; NVIDIA Corporation, Santa Clara, CA, USA) and an Intel Xeon Platinum 8352V CPU (Intel Corporation, Santa Clara, CA, USA) under Ubuntu 22.04. CUDA 12.1 and Python 3.12 are used for training and inference.

The proposed HARM-YOLO11n is trained for 300 epochs from scratch using stochastic gradient descent (SGD) with momentum 0.937 and weight decay $5\times 10^{-4}$. Input images are resized to 640×640, and the batch size is set to 16. The initial learning rate is set to 0.01 with a three-epoch linear warm-up. Automatic mixed precision (AMP) is enabled, and the random seed is fixed to 0 with deterministic algorithmic operations.

Data augmentation consists of offline and online stages. Offline augmentation applies geometric transformations, photometric adjustments, blur and noise operations, and occlusion simulation to each training image up to four times using randomly selected strategies, yielding a five-fold expansion of the original dataset. Online augmentation is applied during training and includes mosaic augmentation (disabled after epoch 10), random horizontal flipping (p=0.5), HSV channel perturbations (H = 0.015, S = 0.7, V = 0.4), random translation (±0.1), random scaling (±0.5), RandAugment, and random erasing (p=0.4).

The classification loss weights are set to 7.5, 0.5, and 1.5 for bounding box regression, classification, and distribution focal loss (DFL), respectively.

For inference, the confidence threshold is set to 0.25, and non-maximum suppression (NMS) is applied with an IoU threshold of 0.5, a maximum of 300 detections per image, and class-agnostic NMS disabled. The frames per second (FPS) metric is measured on the inference stage only (excluding data preprocessing and postprocessing), with a batch size of 1, 30 warm-up iterations, and the average over 3 repeated runs.

For fair comparison, all baseline methods reported in Tables 5–7 are re-trained under the same experimental protocol, using identical training schedules, input resolution, batch size, optimization strategy, and data augmentation settings. Unless otherwise specified, all reported results are obtained under this unified evaluation protocol.

### 4.2. Evaluation Metrics

Detection performance is evaluated using precision (P), recall (R), mean average precision at an IoU threshold of 0.5 (mAP@0.5), and mean average precision averaged over IoU thresholds from 0.5 to 0.95 with a step size of 0.05 (mAP@[0.5:0.95]), following the standard COCO evaluation protocol. While mAP@0.5 reflects overall detection accuracy, mAP@[0.5:0.95] provides a more stringent evaluation of localization quality. In addition, the number of parameters (Params), giga floating-point operations (GFLOPs), model file size (Model/MB), frames per second (FPS), and single-frame inference latency (Latency/ms) are reported to provide a comprehensive assessment of model complexity and inference efficiency.

### 4.3. Ablation Studies

To systematically evaluate the effectiveness of the proposed components, ablation experiments are conducted on the POCO-Dataset using YOLO11n as the baseline detector. SRFE, the SCAM, PAMSF, and IASW are progressively integrated into the baseline model under identical training and evaluation settings. Quantitative results are reported in Table 1, while sensitivity analyses are provided in Table 2 and Table 3.

**Overall results.** As shown in Table 1, all proposed components contribute positively to the final performance. Compared with the baseline YOLO11n, the complete HARM-YOLO11n framework improves mAP1 from 84.4% to 87.5% and mAP2 from 62.3% to 66.5%, corresponding to gains of 3.1 and 4.2 percentage points, respectively. The larger improvement observed under mAP2 indicates that the proposed framework mainly benefits localization-sensitive scenarios involving small-scale and partially occluded targets.

**Effect of structural re-parameterized feature enhancement (SRFE).** After introducing SRFE, mAP2 increases from 62.3% to 63.3% with only a minor increase in model parameters. Since SRFE combines structural re-parameterization and channel recalibration, the improvement is mainly associated with enhanced feature representation under occlusion and incomplete visual observations. The relatively larger gain under localization-sensitive metrics suggests that SRFE primarily improves structural feature extraction rather than confidence estimation.

**Effect of spatial-channel adaptive module (SCAM).** The SCAM increases mAP2 from 62.3% to 64.9%, yielding the largest individual gain among the lightweight feature enhancement modules. This result indicates that selective spatial-channel computation effectively suppresses redundant responses in dense classroom scenes. The simultaneous improvement in precision and recall further suggests that the SCAM enhances feature discrimination while preserving useful contextual information.

**Effect of position-adaptive multi-scale fusion (PAMSF).** PAMSF improves mAP2 from 62.3% to 64.7% and increases recall from 79.7% to 81.3%. Considering the substantial scale variation present in classroom images, the observed improvement is mainly attributed to adaptive cross-scale feature aggregation. The larger gain in recall suggests improved perception of small and distant behavior targets that are difficult to capture using fixed fusion strategies.

**Effect of IoU-adaptive soft weight (IASW).** When applied independently, IASW produces relatively limited performance variation. However, after integration with SRFE, the SCAM, and PAMSF, the complete framework further improves mAP2 from 66.2% to 66.5%. This observation suggests that IASW primarily acts as an optimization-oriented component rather than a feature representation module. By dynamically adjusting sample contributions according to localization quality, IASW helps to stabilize optimization and refine decision boundaries during later training stages.

**Comparison with related loss functions.** To further evaluate the effectiveness of IASW, comparative experiments are conducted with representative training-time weighting and supervision strategies, including focal loss (FL) [34], quality focal loss (QFL) [35], and varifocal loss (VFL) [36]. All loss-function variants are implemented under identical architectural settings, where only the classification and localization supervision strategy is replaced while keeping SRFE, the SCAM, and PAMSF unchanged. The model parameters remain 4.4 M for all variants since the loss formulation does not affect inference-time complexity.

Quantitative results are reported in Table 4. Compared with FL, QFL, and VFL, the proposed IASW achieves the highest mAP and recall across all metrics. Specifically, IASW improves mAP1 by 2.2–2.4 percentage points and mAP2 by 1.0–1.8 percentage points over the compared loss functions. The recall improvement is particularly notable, suggesting that IASW’s localization-aware weighting is more effective in recovering hard positive samples under occlusion and scale variation, where positioning quality varies substantially across anchors.

The performance differences stem from the design choices of each loss function. FL down-weights easy samples via class-prediction confidence, but does not explicitly model localization quality. QFL extends FL by jointly modeling classification and IoU, but uses a fixed weighting scheme that is not adaptive to the batch-wise optimization state. VFL introduces IoU-aware supervision by reweighting positive samples with IoU scores, yet its IoU-weighting is not modulated by batch-level statistics. In contrast, IASW combines localization awareness with dynamic batch-wise thresholding (μ), enabling it to adjust sample emphasis according to the current training state, which is particularly beneficial when annotation quality varies across batches in dense classroom scenes.

**Module coupling analysis.** The contributions of the proposed modules are not strictly additive. Summing the individual mAP2 gains of SRFE (+1.0), the SCAM (+2.6), PAMSF (+2.4), and IASW (−0.3) yields a theoretical improvement of 5.7 percentage points. In contrast, the complete framework achieves an overall gain of 4.2 percentage points. This discrepancy indicates the existence of overlapping feature refinement effects among different modules.

From a functional perspective, SRFE mainly improves representation robustness under occlusion, the SCAM enhances feature selectivity by suppressing redundant responses, PAMSF strengthens multi-scale interaction across pyramid levels, and IASW stabilizes optimization through localization-aware sample weighting. Since these components operate at different stages of the detection pipeline, their effects are complementary rather than fully independent. The observed performance gains therefore arise from coordinated feature refinement and optimization instead of simple accumulation of isolated improvements.

**Efficiency analysis.** The complete framework increases the parameter count from 2.6 M to 4.4 M (approximately 70% relative to the baseline). As reported in Table 1, the majority of this growth is attributable to PAMSF, which introduces parallel fusion pathways across feature pyramid levels to handle multi-scale objects in dense classroom scenes; this structural expansion is the primary cost of the observed gains in localization accuracy (mAP2: +2.4 percentage points from PAMSF alone). Importantly, at 4.4 M parameters, HARM-YOLO11n remains substantially more compact than several detectors with comparable or lower accuracy, including YOLOv9s (7.2 M), YOLOv7-tiny (6.0 M), and YOLOv6s (16.3 M), while maintaining 88.1 FPS—well within the real-time regime. The “lightweight” characterization therefore rests on the overall accuracy–efficiency trade-off rather than on parameter count in isolation: the framework achieves the highest mAP1 and mAP2 among all compared methods in Table 5 at a parameter budget that is lower than most alternatives at similar performance levels, and its inference speed remains sufficient for practical deployment in classroom monitoring scenarios.

**Sensitivity analysis.** To ensure statistical reliability, the PConv ratio experiments are repeated with three different random seeds (0, 42, and 123), and the results are reported as mean ± standard deviation in Table 2. The sensitivity results in Table 2 and Table 3 indicate stable operating regions for both architectural and optimization hyperparameters. For the SCAM, the channel ratio r=1/4 provides the best trade-off between detection performance and model complexity, achieving an mAP@0.5 and mAP@[0.5:0.95] comparable to r=1/2 while reducing parameters by 4.3%, GFLOPs by 4.7%, model size by 3.3%, and latency by 1.7%, with a 2.8% improvement in FPS. For IASW, Δ=0.10 achieves the highest overall detection accuracy, indicating that moderate localization-aware weighting is more effective than overly aggressive or overly conservative reweighting. The low standard deviations in Table 2 further confirm the robustness of the proposed design under different parameter settings.

### 4.4. Comparison with Representative Detection Models

To evaluate the effectiveness of HARM-YOLO11n, comparative experiments are conducted on the POCO-Dataset under identical training settings. The comparison spans diverse detection paradigms. YOLOv12 [40] and YOLOv13 [41] are included as they represent the latest developments in attention-centric and hypergraph-enhanced real-time detection, respectively, offering up-to-date baselines. YOLOv8-Ghost-P2 [42], although originally designed for medical imaging, is adopted here as a lightweight architectural reference: its Ghost-module-based design and dedicated P2 small-object detection head are potentially beneficial for dense small-target scenarios such as classroom behavior detection. Transformer-based detectors, two-stage detectors, and recent classroom-specific methods are also evaluated for completeness.

As reported in Table 5, HARM-YOLO11n achieves 87.5% mAP@0.5 and 66.5% mAP@[0.5:0.95], outperforming YOLO11n by 3.1% and 4.2%, respectively. The proposed method also achieves the highest precision (87.7%) and competitive recall (82.4%) among lightweight detectors while maintaining 88.1 FPS inference speed.

Compared with transformer-based and two-stage detectors, HARM-YOLO11n shows a more favorable balance between accuracy and efficiency. Although some YOLO variants achieve higher inference speed, their performance under stricter localization metrics is lower.

Compared with YOLO11n, the increase in computational cost (2.6 M to 4.4 M parameters) is accompanied by consistent improvements in both detection accuracy and recall, indicating an improved accuracy–efficiency trade-off under the evaluated settings.

**Table 5:** Comparison with representative detection models on the POCO-Dataset. *Note:* mAP1 denotes mAP@0.5, while mAP2 denotes mAP@[0.5:0.95].

Models	P/%	R/%	mAP1	mAP2	Params	FPS
YOLOv13n [41]	87.1	79.0	85.1	63.7	2.5 M	43.4
YOLOv12n [40]	83.7	79.1	83.7	62.1	2.5 M	60.3
YOLOv11n [15]	84.1	79.7	84.4	62.3	2.6 M	112.5
YOLOv10n [43]	82.6	79.1	83.6	61.9	2.3 M	95.7
YOLOv9s [44]	87.5	80.9	86.2	66.2	7.2 M	46.9
YOLOv8n [14]	85.1	78.3	84.1	62.2	3.0 M	148.1
YOLOv7-tiny [45]	86.1	82.2	82.8	54.9	6.0 M	243.9
YOLOv6s [46]	87.6	81.1	86.3	65.7	16.3 M	104.9
YOLOv5n [13]	84.1	78.1	83.5	61.0	2.5 M	129.7
RT-DETR [47]	76.0	76.7	80.0	53.9	31.6 M	24.6
Faster R-CNN [21]	74.9	54.7	56.5	27.2	137.1 M	11.7
YOLOv8-Ghost-P2 [42]	85.8	81.7	86.6	63.8	1.6 M	95.9
Wang et al. [23] (2023)	83.3	79.4	84.2	61.2	2.6 M	102.1
Tang et al. [22] (2022)	84.9	77.2	83.6	61.1	2.4 M	111.8
SmartLite-YOLO [30]	84.2	75.7	82.3	60.9	1.6 M	138.7
YOLOv8n_BT [31]	87.4	81.8	87.1	65.9	3.0 M	148.3
YOLO-CBR [32]	85.1	83.5	86.9	65.4	3.0 M	87.1
**HARM-YOLO11n (Ours)**	**87.7**	82.4	**87.5**	**66.5**	4.4 M	88.1

**Figure 9:**
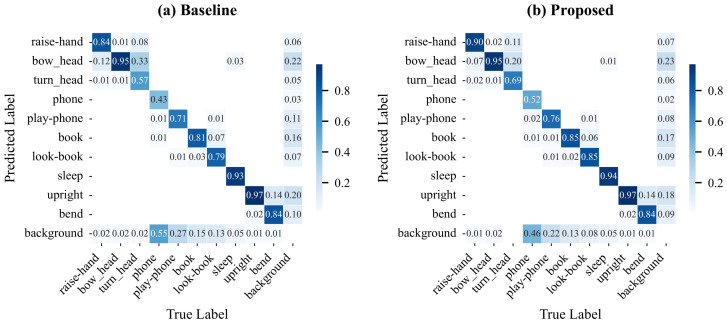
Normalized confusion matrices of YOLO11n (**a**) and HARM-YOLO11n (**b**) on the POCO-Dataset.

Figure 9 presents confusion matrices on the POCO-Dataset. HARM-YOLO11n reduces inter-class confusion in visually similar categories such as *raise-hand* and *play-phone*, indicating improved discriminative capability in dense classroom scenes.

**Comparison with classroom-specific methods.** Among classroom-specific detectors, YOLOv8n_BT and YOLO-CBR are the most competitive baselines. YOLOv8n_BT [31] achieves 87.1% mAP1 and 65.9% mAP2 through bi-level routing attention, yet its design remains focused on architectural enhancement alone. YOLO-CBR [32] attains 86.9% mAP1 and 65.4% mAP2 using dual-stage routing attention on YOLOv11n, but does not address optimization-level coupling. HARM-YOLO11n surpasses both by 0.4–0.6 percentage points in mAP1 and 0.6–1.1 percentage points in mAP2, while also achieving higher recall. SmartLite-YOLO [30] maintains the smallest parameter count (1.6 M) but yields the lowest mAP2 (60.9%) among classroom-specific methods, suggesting that aggressive lightweight design may compromise localization quality. Tang et al. [22] and Wang et al. [23] similarly lag in mAP2 (61.1% and 61.2%), as they rely solely on attention-enhanced detection without jointly optimizing feature fusion and training objectives. The consistent margin over these methods—particularly under the stricter mAP2 metric—suggests that the unified treatment of representation, fusion, and optimization in HARM-YOLO11n provides complementary benefits beyond single-dimension improvements.

Overall, the results show consistent improvements over representative methods while maintaining real-time performance constraints.

### 4.5. Qualitative Analysis

Qualitative analysis is conducted on representative samples from the POCO-Dataset under dense scenes, occlusion, scale variation, and background clutter.

**Figure 10:**
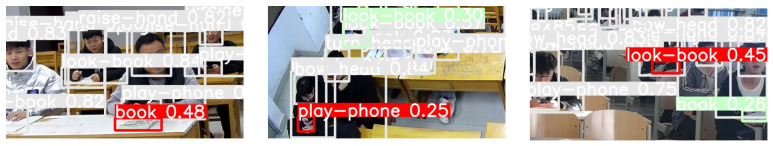
Qualitative detection results on representative classroom scenes. Light gray boxes: detected by all three models; light green boxes: missed by SmartLite-YOLO but detected by YOLO11n and HARM-YOLO11n; red boxes: detected only by HARM-YOLO11n.

As illustrated in Figure 10, HARM-YOLO11n produces more complete object coverage in crowded classroom scenarios. Compared with SmartLite-YOLO and YOLO11n, additional small-scale or partially occluded instances are detected, particularly in densely populated regions.

**Figure 11:**
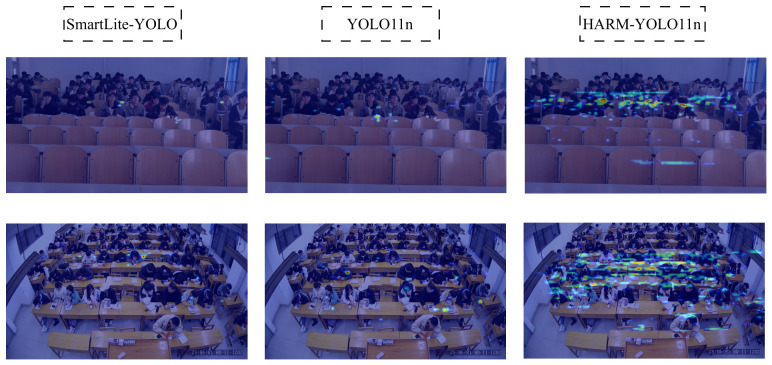
Grad-CAM visualization comparison among SmartLite-YOLO, YOLO11n, and HARM-YOLO11n. From left to right: SmartLite-YOLO, YOLO11n, and HARM-YOLO11n.

Figure 11 presents Grad-CAM visualizations under identical input samples. HARM-YOLO11n exhibits more spatially distributed activation patterns over target regions, especially under occlusion and crowding conditions, indicating improved utilization of contextual information.

However, residual background activations can still be observed in highly cluttered scenes, suggesting that feature ambiguity remains under severe occlusion and strong background similarity.

### 4.6. Multi-Dataset Evaluation

To evaluate the robustness of HARM-YOLO11n under heterogeneous classroom environments, multi-dataset experiments are conducted on SCB-Dataset3 and STBD-08 using identical training settings as described in Section 4.1. These datasets differ in annotation granularity, scene density, and behavioral distribution.

**Results on SCB-Dataset3.** As reported in Table 6, HARM-YOLO11n achieves 72.2% mAP@0.5 and 57.2% mAP@[0.5:0.95], outperforming YOLO11n by 4.1% on both metrics. The model also achieves higher recall (71.0%) while maintaining real-time inference speed (89.6 FPS).

**Results on STBD-08.** As shown in Table 7, HARM-YOLO11n achieves 92.1% mAP@0.5 and 79.3% mAP@[0.5:0.95], with improvements of 1.0% and 1.4% over YOLO11n, respectively.

## 5. Discussion

Experimental results across all datasets indicate that HARM-YOLO11n shows consistent performance under real-time constraints for dense classroom behavior detection.

The observed improvements are more evident under mAP@[0.5:0.95], suggesting that the proposed framework is primarily associated with enhanced localization quality in scenarios involving small-scale and partially occluded objects.

From a functional perspective, SRFE enhances structural feature representation under occlusion, the SCAM improves channel-wise feature selection in cluttered scenes, and PAMSF facilitates adaptive cross-scale feature interaction. IASW introduces localization-aware sample weighting, which is associated with more stable optimization behavior during training.

The ablation results indicate that these modules provide complementary effects across representation learning, feature aggregation, and optimization stages. The resulting performance gains are therefore associated with coordinated feature refinement rather than any single component.

Although additional adaptive modules increase the parameter count, the framework maintains real-time inference capability at 88.1 FPS. The relationship between parameter count and inference speed is not strictly linear: PAMSF adds parameters through parallel fusion branches that introduce minimal sequential overhead, while the SCAM’s partial convolution mechanism reduces per-channel computation. In terms of practical lightweight deployment metrics—model size (8.8 MB), GFLOPs (12.3), and latency (11.4 ms)—the framework remains suitable for resource-constrained educational environments.

Several limitations remain. The current framework does not explicitly model temporal information and therefore cannot exploit video-level behavioral consistency. In addition, performance may still be affected by substantial domain shifts caused by differences in classroom layouts and annotation styles. Moreover, all inference-time measurements were conducted on a workstation-grade GPU (RTX 4090); on-device benchmarking on edge platforms such as Jetson, embedded GPU/NPU, or CPU-only environments has not yet been performed, which would provide a more direct validation of real-world deployability in resource-constrained educational settings. Future work will investigate lightweight temporal modeling, edge-device deployment validation, and domain-adaptive feature alignment strategies.

We also note that the three datasets used in this study are publicly available academic benchmarks collected for classroom behavior analysis, and the present work does not involve direct collection of student data. Nevertheless, vision-based classroom monitoring raises ethical and privacy concerns. The original datasets were anonymized where feasible, but facial features may still be present in some frames. Practitioners should ensure compliance with applicable data protection regulations, deploy on-device processing where possible, and adopt transparent disclosure practices regarding system deployment and data retention. Detection outputs should serve as auxiliary teaching feedback rather than for evaluative or disciplinary purposes.

## 6. Conclusions

This paper proposes HARM-YOLO11n, a lightweight framework for dense classroom behavior detection that integrates SRFE, an SCAM, PAMSF, and IASW.

Experimental results on POCO-Dataset, SCB-Dataset3, and STBD-08 indicate that the proposed framework achieves improved detection performance compared with representative baseline detectors while maintaining real-time inference efficiency. Ablation and multi-dataset evaluations further demonstrate the contribution of individual components under different classroom scenarios.

Overall, the results suggest that jointly optimizing feature representation, multi-scale interaction, and localization-aware learning provides an effective solution for dense classroom behavior detection in resource-constrained educational environments. Future work will explore temporal behavior modeling and domain-adaptive learning to further improve robustness across diverse classroom settings.

## Figures and Tables

**Table 1:** Ablation study of individual components on the POCO-Dataset. *Note:* mAP1 denotes mAP@0.5, while mAP2 denotes mAP@[0.5:0.95], averaged over IoU thresholds from 0.5 to 0.95 with a step size of 0.05.

SRFE	SCAM	PAMSF	IASW	P/%	R/%	mAP1	mAP2	Params	FPS
				84.1	79.7	84.4	62.3	2.6 M	112.5
✓				86.0	79.6	85.0	63.3	2.7 M	106.3
	✓			86.2	81.2	86.0	64.9	2.9 M	114.9
		✓		86.0	81.3	85.7	64.7	4.0 M	91.6
			✓	84.2	79.3	84.7	62.0	2.6 M	115.6
✓	✓			87.2	79.9	85.9	65.1	3.4 M	116.9
✓	✓	✓		86.4	82.4	86.8	66.2	4.4 M	90.0
✓	✓	✓	✓	**87.7**	**82.4**	**87.5**	**66.5**	4.4 M	88.1

**Table 2:** Sensitivity analysis of the PConv channel ratio *r* on the POCO-Dataset. *Note:* mAP1 denotes mAP@0.5, while mAP2 denotes mAP@[0.5:0.95]. All metrics are averaged over three runs with random seeds 0, 42, and 123, reported as mean ± standard deviation.

*r*	P/%	R/%	mAP1	mAP2	Params	GFlops	Model	FPS	Latency
1/2	88.4 ± 0.8	82.4 ± 0.4	87.4 ± 0.1	67.1 ± 0.2	4.6 M	12.9	9.1 M	85.7	11.6 ms
1/4	87.1 ± 0.6	**83.2** ± 0.8	**87.4** ± 0.1	66.9 ± 0.5	**4.4 M**	12.3	8.8 M	88.1	11.4 ms
1/8	87.9 ± 1.0	81.8 ± 1.5	87.0 ± 0.6	66.6 ± 0.5	4.4 M	12.1	8.7 M	90.1	11.1 ms

**Table 3:** Sensitivity analysis of the IASW margin parameter Δ on the POCO-Dataset. *Note:* Δ denotes the margin parameter in the IoU-adaptive soft weight (IASW) strategy. mAP1 denotes mAP@0.5, while mAP2 denotes mAP@[0.5:0.95].

Δ	P/%	R/%	mAP1	mAP2	Params
0.05	88.0	81.2	86.6	66.2	4.4 M
0.10	87.7	**82.4**	**87.5**	**66.5**	**4.4 M**
0.15	87.8	80.9	86.5	66.5	4.4 M
0.20	88.1	77.7	84.6	65.0	4.4 M

**Table 4:** Comparison of IASW with representative loss functions on the POCO-Dataset. All variants share the same architecture (SRFE + SCAM + PAMSF) and differ only in the training-time supervision strategy. *Note:* mAP1 denotes mAP@0.5, while mAP2 denotes mAP@[0.5:0.95].

Loss Function	P/%	R/%	mAP1	mAP2	Params
Focal Loss [34]	82.7	80.4	85.3	65.5	4.4 M
Quality Focal Loss [35]	84.1	77.6	85.1	64.7	4.4 M
Varifocal Loss [36]	85.3	78.2	85.1	65.1	4.4 M
**IASW**	**87.7**	**82.4**	**87.5**	**66.5**	**4.4 M**

**Table 6:** Multi-dataset comparison on SCB-Dataset3. *Note:* mAP1 denotes mAP@0.5, while mAP2 denotes mAP@[0.5:0.95].

Models	P/%	R/%	mAP1	mAP2	Params	FPS
YOLOv13n [41]	59.7	69.2	66.4	50.6	2.5 M	43.8
YOLOv12n [40]	63.7	68.0	67.9	52.8	2.5 M	60.4
YOLOv11n [15]	63.1	69.3	68.1	53.1	2.6 M	106.3
YOLOv10n [43]	64.3	66.7	66.7	52.0	2.3 M	107.1
YOLOv8n [14]	66.5	66.7	69.0	53.6	3.0 M	159.2
YOLOv5n [13]	65.5	67.6	68.1	52.3	2.5 M	140.9
RT-DETR [47]	69.8	62.6	67.6	52.0	31.6 M	26.2
YOLOv8-Ghost-P2 [42]	59.9	65.0	63.0	47.1	1.6 M	106.1
**HARM-YOLO11n (Ours)**	**67.2**	**71.0**	**72.2**	**57.2**	4.4 M	89.6

**Table 7:** Multi-dataset comparison on STBD-08 dataset. *Note:* mAP1 denotes mAP@0.5, while mAP2 denotes mAP@[0.5:0.95].

Models	P/%	R/%	mAP1	mAP2	Params	FPS
YOLOv13n [41]	87.3	87.0	90.7	77.6	2.5 M	42.7
YOLOv12n [40]	86.7	87.2	91.0	77.7	2.5 M	60.4
YOLOv11n [15]	87.9	86.3	91.1	77.9	2.6 M	110.6
YOLOv10n [43]	89.5	84.5	90.6	77.4	2.3 M	110.2
YOLOv8n [14]	87.3	86.7	91.2	77.6	3.0 M	157.4
YOLOv5n [13]	87.2	86.4	91.1	77.0	2.5 M	139.5
RT-DETR [47]	80.9	83.2	85.8	69.5	31.6 M	27.1
YOLOv8-Ghost-P2 [42]	85.2	85.1	89.3	75.1	1.6 M	102.4
**HARM-YOLO11n (Ours)**	87.9	**88.4**	**92.1**	**79.3**	4.4 M	90.2

## Data Availability

Data are available upon request due to privacy or ethical restrictions. Data from this study are available from the corresponding authors upon request. Because of the privacy implications of the data in this study, these data are not publicly available.
